# Augmenting Telepostpartum Care With Vision-Based Detection of Breastfeeding-Related Conditions: Algorithm Development and Validation

**DOI:** 10.2196/54798

**Published:** 2024-06-24

**Authors:** Jessica De Souza, Varun Kumar Viswanath, Jessica Maria Echterhoff, Kristina Chamberlain, Edward Jay Wang

**Affiliations:** 1 Department of Electrical and Computer Engineering University of California, San Diego La Jolla, CA United States; 2 Department of Computer Science and Engineering University of California, San Diego La Jolla, CA United States; 3 Division of Extended Studies University of California, San Diego La Jolla, CA United States

**Keywords:** remote consultations, artificial intelligence, AI for health care, deep learning, detection model, breastfeeding, telehealth, perinatal health, image analysis, women’s health, mobile phone

## Abstract

**Background:**

Breastfeeding benefits both the mother and infant and is a topic of attention in public health. After childbirth, untreated medical conditions or lack of support lead many mothers to discontinue breastfeeding. For instance, nipple damage and mastitis affect 80% and 20% of US mothers, respectively. Lactation consultants (LCs) help mothers with breastfeeding, providing in-person, remote, and hybrid lactation support. LCs guide, encourage, and find ways for mothers to have a better experience breastfeeding. Current telehealth services help mothers seek LCs for breastfeeding support, where images help them identify and address many issues. Due to the disproportional ratio of LCs and mothers in need, these professionals are often overloaded and burned out.

**Objective:**

This study aims to investigate the effectiveness of 5 distinct convolutional neural networks in detecting healthy lactating breasts and 6 breastfeeding-related issues by only using red, green, and blue images. Our goal was to assess the applicability of this algorithm as an auxiliary resource for LCs to identify painful breast conditions quickly, better manage their patients through triage, respond promptly to patient needs, and enhance the overall experience and care for breastfeeding mothers.

**Methods:**

We evaluated the potential for 5 classification models to detect breastfeeding-related conditions using 1078 breast and nipple images gathered from web-based and physical educational resources. We used the convolutional neural networks Resnet50, Visual Geometry Group model with 16 layers (VGG16), InceptionV3, EfficientNetV2, and DenseNet169 to classify the images across 7 classes: healthy, abscess, mastitis, nipple blebs, dermatosis, engorgement, and nipple damage by improper feeding or misuse of breast pumps. We also evaluated the models’ ability to distinguish between healthy and unhealthy images. We present an analysis of the classification challenges, identifying image traits that may confound the detection model.

**Results:**

The best model achieves an average area under the receiver operating characteristic curve of 0.93 for all conditions after data augmentation for multiclass classification. For binary classification, we achieved, with the best model, an average area under the curve of 0.96 for all conditions after data augmentation. Several factors contributed to the misclassification of images, including similar visual features in the conditions that precede other conditions (such as the mastitis spectrum disorder), partially covered breasts or nipples, and images depicting multiple conditions in the same breast.

**Conclusions:**

This vision-based automated detection technique offers an opportunity to enhance postpartum care for mothers and can potentially help alleviate the workload of LCs by expediting decision-making processes.

## Introduction

### Background

The benefits of breastfeeding for both the mother and baby, such as lower gastrointestinal infections in the child, more rapid maternal weight normalization after birth, and prolonged amenorrhea for the mother, are just a few examples of why physicians recommend breastfeeding for at least 6 months [1-5]. Breastfeeding rates are on the rise in the United States, with 83.2% of newborn infants being breastfed in 2019, thanks to increased education and promotion of its benefits [6]. Despite the compelling evidence, many families struggle to continue breastfeeding. Although 95% of mothers initiate breastfeeding, the continuation rate drops to <41% and <19% for exclusive breastfeeding at 3 and 6 months, respectively [7]. Parents who breastfeed may face issues, such as low milk supply, fatigue, medical problems, difficulties with feeding techniques or pain, and lack of social support [8-10].

Lactation consultant (LC) professionals specialize in breastfeeding, milk supply, breast and nipple issues, breast milk management, and prenatal education. LCs ensure a mother’s smooth and painless transition into breastfeeding and increase the possibility of continued breastfeeding through 6 months or longer [11,12]. The availability of international board-certified LCs (IBCLCs) globally is limited. In 2021, there were 3.6 million births in the United States and only 18,500 LCs with IBCLC certification, a rate of 194 babies per LC a year. In low- and middle-income countries such as Brazil, for instance, there were 2.6 million births in the same year but only 154 certified LCs, resulting in a rate of 16,883 babies per LC per year. The high demand for LCs, coupled with geographic and financial barriers, underscores the need for better tools to improve access to specialized lactation services, especially in less urbanized areas where such resources are scarce, leading to decreased breastfeeding support [13-20].

Another issue is professional availability itself, as LCs often combine their practice with midwife nursing, splitting their time between prenatal visits, attending births, lactation consultations, and managing their patients, which can lead to professional exhaustion, burnout, and emotional stress [21-23]. Moreover, the predominantly independent practice of LCs outside the United States, without the support of clinics with sophisticated patient management and triage systems, further complicates their time management and patient organization [22,24].

### Supporting LCs Through Tele-Lactation Services

Tele-lactation services facilitate text, audio, and video communication. This enables LCs to consult with patients from any location, reduces travel time, helps balance their workload, increases their availability to receive new patients, and provides quicker responses to their patients [20]. Complementing tele-lactation services, patient triaging using information systems allow LCs to prioritize in-person visits for severe cases requiring physical assessment, while less critical cases can be handled remotely [25,26]. Prior research suggests that LCs would benefit from time-saving tools for efficient patient information delivery while focusing on mitigating prolonged interactions, helping alleviate the burden on these professionals with a load of patients [22,27]. As LCs often follow up with their patients up to weeks after birth to ensure positive breastfeeding outcomes, an easy-to-access system to monitor patient progress is essential for effective patient triage, facilitating consultation scheduling, holding remote consultations, or providing reassurance. However, LCs’ current access to remote consultation systems lacks patient triaging tools and is not time efficient, indicating an area in need of development.

Our work proposes a novel method for the identification of breastfeeding-related conditions using convolutional neural networks (CNNs). We evaluated a self-curated data set containing 7 different breastfeeding conditions on 5 distinct CNN models. The assessment of breast conditions is vital as pain and discomfort experienced during breastfeeding is a major barrier faced by parents who want to continue breastfeeding their child. About 80% of mothers are estimated to experience nipple pain and fissures, while 20% are estimated to experience mastitis [28,29]. Our pipeline incorporates automatic detection of visually discernible painful breastfeeding-related conditions, such as nipple cracks and fissures related to poor latching and positioning; skin conditions, such as dermatitis, eczema, thrush, or herpes; and risk of mastitis spectrum issues, such as engorgement, abscess, and nipple blebs. The CNN model is used for automatic detection of breast conditions, which can benefit the triaging of remote lactation patients for faster and more efficient patient response based on their conditions.

Our work evaluated 5 distinct CNN models’ ability to differentiate between healthy and various unhealthy breast conditions (including breast abscesses, dermatoses, engorgement, mastitis, nipple blebs, and nipple damage) by performing both multiclass and binary evaluations on 1078 breast images. We evaluated the model’s performance using the data set with and without data augmentation techniques. The data were divided into training, validation, and testing sets, using k-fold cross-validation for robustness. Performance evaluation on the best model includes an average area under the curve (AUC) of 0.93 for all conditions after data augmentation and precise detection of healthy breasts (precision of 84.4%) and unhealthy breasts (average precision of 66%, SD 12.8%) for 6 conditions. For binary classification, we achieved, with the best model, an average AUC of 0.96 for all conditions after data augmentation and precise detection of healthy breasts (precision of 93.8%) and unhealthy breasts (precision of 83.5%). The breast images have been curated from perinatal education resources such as images and video recordings under various lighting, environments, and image-taking conditions, where we examined potential issues around how the images are taken and their impacts on performance. Finally, we provide insights into future designs of user interfaces and guidance needed for the proper application of the system.

### Related Work

#### Lactating Care Pipeline: In-Person, Remote, and Hybrid

Health care providers introduce breastfeeding options to expectant mothers, including educational materials in print or web-based, during prenatal care. The initiation of breastfeeding after delivery is timed according to the type of birth. Many hospitals worldwide follow the United Nations Children’s Fund and World Health Organization baby-friendly initiative, prioritizing maternal and infant health and supporting mothers facing challenges [30,31]. After a child’s birth, families often seek breastfeeding support from LCs, who typically offer hands-on consultations from birth until support is no longer required [18]. They conduct visual and physical evaluations of both mother and baby, assessing the baby’s internal mouth structure, breast and nipple anatomy, and milk supply and ensuring proper attachment or repositioning of the baby to prevent nipple fissures. LCs may also introduce laser therapy as a treatment option for damaged nipples from breast pump misuse or issues with baby attachment [8]. The immersive approach of LCs is crucial for providing personalized and effective lactation support to mothers and infants.

#### Remote Lactation Care

The widespread adoption of smartphone communication apps, particularly WhatsApp (Meta Platforms, Inc), has transformed public health facilities, including family clinics in limited-income countries, offering various patient services such as appointment scheduling, health guidance, and vaccine campaign notifications [32-34]. WhatsApp has become a popular communication tool between LCs and patients, facilitating breastfeeding education and family support during the neonatal period [35,36]. During the COVID-19 pandemic, LCs transitioned to telehealth consultations using established smartphone apps such as WhatsApp, Instagram (Meta Platforms, Inc), and Facebook (Meta Platforms, Inc). LCs adapted their approach to maintain quality care despite resource limitations in remote consultations [37,38]. Similar to other practices requiring physical evaluation, LCs reimagined their methods when shifting from in-person to remote consultations, using communication and social media apps to reach and educate parents while having broader visibility in their community [37,39].

Remote lactation care presents challenges, including limited visibility during video calls, communication difficulties, and technical issues [18,40,41]. Despite challenges, remote care offers benefits, reducing the mother’s sense of isolation, enabling faster feedback, and promoting effective communication and patient engagement for improved independent learning [17,18,22]. These benefits positively impact mothers’ intentions in exclusive breastfeeding for up to 6 months and reduce the risk of breastfeeding cessation at 3 months by 25% [42].

#### Hybrid Lactation Care

Previous research showed that fully remote consultations work well for cases where geographic distance, transportation issues, or patient disease prevent in-person meetings between patients and providers. LCs often conduct remote consultations from their workplaces, including personal offices, clinics, or hospitals, especially when they are also midwives with on-call responsibilities [37]. They provide consultations for patients before birth, after birth, and in emergency cases where the mother is facing breastfeeding challenges [22]. Depending on the nature of the consultation, in-person or remote visits are chosen to meet the patient’s specific needs. In summary, remote care complements in-person care, being a valuable resource for mothers seeking guidance, reassurance, and confidence, particularly in the absence of a supportive home environment [38].

LCs, especially those who are also midwives, have limited time availability due to demanding schedules and receiving numerous remote messages from patients daily, some requiring higher priority attention [22,43]. Manually sorting through patient messages to determine priority can be time consuming and inconvenient for mothers with urgent needs. Our work proposes a computer vision–based system to triage breast conditions, facilitating telehealth and assisting LCs in identifying patients who require immediate responses in remote settings.

### Issues Associated With Breastfeeding

Breastfeeding pain is one of the reasons associated with breastfeeding cessation, which can be caused by issues such as poor attachment of the baby onto the breast, physical conditions of the mother or baby, misuse of breast pumps, oversupply of breast milk, and even environmental conditions [44]. These issues, if left untreated in the first few days after birth, can persist for weeks and pose a threat to breastfeeding continuity beyond 6 months. Some conditions can be fully mitigated when the mother receives orientation and education on the topic. In contrast, other conditions can be alleviated and managed for a better experience for the mother in the case of physical conditions, including nipple physiology, baby tongue-tie, jaw clenching, and excessive milk supply [28,45].

This study concentrates on conditions leading to breastfeeding pain and potential interruption. The first condition is the mastitis spectrum disorder, where about 20% of mothers who breastfeed may face it during their time breastfeeding. This disorder starts with the overproduction of milk and breast engorgement, which can cause milk passage obstruction in the form of galactoceles and nipple blebs. When not properly treated, a case of milk bleb or galactocele can evolve into phlegmon, bacterial, or inflammatory mastitis, which may require patients to treat it with medications and sometimes medical procedures to drain the inflammation fluids from the breast in case it becomes an abscess [46,47]. Conditions associated with mastitis are painful and include symptoms such as redness in the breast, influenza-like symptoms, hardened skin surface in the location of the milk blockage, formation of blisters in the nipple, and even blood in the milk [29,48].

The second condition is nipple damage caused by improper latching and positioning from the infant, excessive pressure from breast pumping devices, infant tongue-tie or palate abnormality, infant’s arrhythmic milk expression, and even infant biting or jaw clenching [9,44]. Considering the cause of nipple damage, 80% of mothers are expected to face some level of nipple issues during breastfeeding, which, if not treated, may cause an average of 35% of these mothers to cease breastfeeding before 1 month [28,45]. Nipple damage is painful and may be visible or invisible. When visible, it can present features at the skin surface, such as fissures, cracks, pus, blood, scarring, or crusting. Some skin dermatoses, such as thrush, herpes, eczema, and psoriasis, are also responsible for discomfort and pain during breastfeeding. These conditions can be caused by friction, weather, and temperature changes and using medications or ingredients that can make the skin prone to these disorders. Dermatoses conditions present on both breast and nipple and can have visible features such as scarring, crusting formations, redness, and thickened skin regions [44]. Our research incorporates breast and nipple images from the following disorders: breast abscess, dermatoses, breast engorgement, inflammatory and bacterial mastitis, nipple blebs, and nipple damage.

### Current Research Supporting Lactating Mothers

Extensive literature has highlighted the efficacy of deep learning in assessing breast images, helping detect malignant and benign breast tumors for both lactating and nonlactating women [49-54]. This has helped improve the precision of breast ultrasound and mammogram examinations, involving the use of medical imaging previously taken in medical facilities to enhance the evaluation of breast-related illnesses and allow better accuracy in diagnosis for medical personnel [53]. However, these studies relied on images gathered from specialized equipment found only in health care facilities. They did not extend their evaluation to external body images, focusing primarily on aiding health care practitioners in diagnosis. Our work diverges from previous contributions by primarily focusing on using external breast images gathered from personal devices, such as smartphones or cameras from lactating patients, to identify breastfeeding-related conditions in the early stages and evaluate the necessity of further examination and medical intervention.

In the context of breastfeeding disorders, there is a lack of research regarding using deep learning algorithms to evaluate real breast images and identify abnormalities such as mastitis, nipple fissures, dermatoses, and abscesses. To illustrate, literature addressing the early prediction of mastitis mainly originates from agricultural studies, in which the risk of mastitis is constantly assessed to prevent a reduction in animal milk production, which significantly impacts the dairy industry [55,56]. This shows a need for research to adapt these technologies for detecting and preventing breastfeeding disorders in humans. Our study is crucial in settings where access to medical professionals and LCs is limited, as it can help prevent breastfeeding cessation, promote maternal-infant bonding, and improve the overall health and well-being of mothers and infants.

## Methods

In this section, we detail the data set collection process, including inclusion and exclusion criteria, data sources, and the characteristics of the images. The section also discusses the artificial intelligence (AI) algorithms used in the study, including the models and their training and validation process, and performance metrics used during evaluation.

### Ethical Considerations

This study was approved by the University of California, San Diego Institutional Review Board (801,904). We did not incorporate any personally identifiable data from the participants into this research.

### Data Set Collection

#### Overview

This study used a breast image data set (refer to Textbox 1 and Table 1), a compilation of physical and digital images specifically curated to train and validate our deep learning model’s ability to distinguish between healthy and unhealthy lactating breasts. The data set includes images categorized according to their respective conditions: healthy lactating breast; nipple injuries due to various causes; nipple blebs due to plugged ducts; breast or nipple with signs of dermatoses; and breasts with engorgement, mastitis, or abscess.

**Data set description.**

**Description**

- Data set size
  393.7 MB (each image: minimum 0.015, average 0.360, and maximum 3.575 MB)
- Dimensions (pixels)
  Width (minimum 68, average 606, and maximum 2448)
  Height (minimum 68, average 607, and maximum 2448)
- Number of images
  1078
- Number of classes
  7
- Number of unique subjects
  586
- Number of images per class
  Abscess: 115
  Dermatoses: 123
  Engorgement: 63
  Mastitis: 180
  Nipple bleb: 82
  Nipple damage: 197
  Healthy: 318
- Visual features per class
  Abscess: swelling and redness, area with palpable fluid collection, and pus
  Dermatoses: rash, discoloration, flaky skin, uneven skin tone, crusting, and redness
  Engorgement: swelling, redness, skin stretched and shiny, and enlarged nipple
  Mastitis: red patches on breast or nipple, swelling, and pus or blood discharge
  Nipple bleb: small white or yellow bumps on nipple or areola, similar to a blister
  Nipple damage: nipple swelling, redness, peeling or flaking skin, bleeding, and shape differences
  Healthy: regular breast and nipple color, may have visible veins
- Number of images per source
  Physical: 178 (eg, books, magazines, and articles)
  Physician websites: 366
  YouTube: 65 (eg, educational channels on women’s health)
  Other: 469 (eg, received by lactation consultants; international board-certified lactation consultant’s Instagram, Google Images, and Flickr; support groups mediated by lactation consultants on social media; and other educational websites)

**Table 1:** Number of images per skin tone per class (FST^a^ [57]).

Class name	FST I	FST II	FST III	FST IV	FST V	FST VI	Not classified^b^
Abscess	28	35	20	8	14	8	2
Dermatoses	17	37	48	13	3	3	2
Engorgement	4	6	18	30	4	0	1
Mastitis	44	69	51	11	1	4	0
Nipple bleb	9	16	18	8	6	3	22
Nipple damage	40	59	22	15	11	5	45
Healthy	61	90	92	21	28	21	5
Total per FST	203	312	269	106	67	44	77

^a^FST: Fitzpatrick skin type.

^b^Not classified due to the absence of breast tissue around the nipple in the image.

#### Data Inclusion and Exclusion Criteria

To be included in the data set, images must meet the following criteria: (1) the image must be in red, green, and blue (RGB) format, either as PNG or JPEG; (2) it must visually have at least 1 of the 7 conditions; (3) the breast or nipple should be visible; (4) the image should be hosted in a trustworthy source (ie, from medical professionals such as physicians, midwife nurses, and IBCLCs), in which the image must have a word or description identifying its condition among the 7 classes to be included as its label; and (5) the visual condition present in the image and the label provided describing the condition should match. Images were excluded from the data set if (1) the breast or nipple were from nonlactating female patients; (2) the condition described on the label and the visual features of the image did not match; (3) the breast or nipple was not visible in the image; and (4) the image did not have any label describing it. A board-certified nurse practitioner (ie, Certified Nurse Practitioner, Advanced Registered Nurse Practitioner, or IBCLC) with >15 years of experience performed a final review of the data set to ensure that images and labels had no discrepancies.

#### Data Source

We collected images from diverse sources such as breastfeeding-related books, articles, web-based blogs for mothers and physicians, YouTube videos from educative organizations, and social media platforms (eg, Instagram, Facebook, and Twitter) of certified health care providers who would have educative resources for mothers. To ensure diversity in geographic and racial representation, we conducted image searches using multiple languages (eg, English, Portuguese, Spanish, French, and Chinese) and used search engines adjusted for other countries.

**Figure 1:**
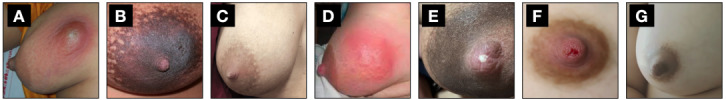
Example images from the testing set that were correctly classified and show features of each breastfeeding-related condition: (A) abscess, (B) dermatoses, (C) engorgement, (D) mastitis, (E) nipple bleb, (F) nipple damage, and (G) healthy.

The images were obtained from a diverse group of female patients with several skin colors and breast and nipple sizes, with unstandardized image sizes, orientations, backgrounds, and light sources. In total, the data set consisted of 1078 images, with 318 images of healthy breasts, 115 images of breast abscesses, 123 images of dermatoses, 63 images of breast engorgement, 180 images of mastitis, 82 images of nipple blebs, and 197 images of nipple damage. As shown in Figure 1 and Table 1, a healthy lactating breast presented a uniform color, was free of redness, and had no signs of discharge. Nipples were expected to exhibit a variety of shapes, including flat, protruded, or inverted, and to vary in size. In engorgement, images showed breast and nipple swelling, skin stretched and shiny, and some light redness due to high milk production. For nipple blebs or nipple damage, signs of laceration, blood, blisters, and redness were expected. Mastitis showed swelling, redness, and discharge of pus or blood in the nipple. Abscess shared similarities with mastitis but involved worsened redness and pus in the infected region and may display signs of rupture. Finally, dermatosis images contained signs of skin rash, breast or nipple uneven skin tone, and crusting.

### AI Algorithms

We examined the performance of 5 CNNs commonly used in computer vision problems: Visual Geometry Group model with 16 layers (VGG16) [58], Resnet50 [59], InceptionV3 [60], EfficientNetV2 [61], and DenseNet169 [62]. All models were built with the PyTorch library for image classification, in which the models had all layers frozen except for the last layer, which was replaced with a fully connected layer adapted to the number of classes—2 for binary classification and 7 for the multiclass task. All models were trained for 100 epochs using the AdamW optimizer with a learning rate of 3e-4, weight decay of 0.1, and batch size of 20. We chose 100 epochs because it was a converging point where the accuracy no longer increased or decreased. For the loss functions, we applied Binary Cross-Entropy with Logits Loss for binary classification tasks, and for multiclass tasks, we used Cross-Entropy Loss, both fine-tuned with class weights to strategically adjust for class imbalances by proportionally penalizing misclassifications in less represented classes. These models were evaluated using stratified k-fold cross-validation with 10 folds. To ensure the robustness of our cross-validation process, we reset any learned parameters by initializing the models from scratch at the beginning of each fold. Instead of using the entire image data set to train the model, we did feature extraction to optimize the training process (detailed in the Feature Extraction section). We compared the performance of the 5 models across the same data and keep the hyperparameters the same: learning rate, weight decay, batch size, and number of epochs.

### Data Set Preprocessing

Before using the images as inputs for the deep learning models, the images were manually cropped to ensure they were deidentified and had no irrelevant content, such as unrelated body areas, clothes, jewelry, identifiable tattoos, or backgrounds, enhancing the model’s accuracy and performance. The images were cropped in a 1:1 ratio to prevent image flattening or warping during resizing and loss of important features. Most images have breast and nipple tissue concentrated in the center of the image, thereby focusing the model’s evaluation on the most relevant areas. Our image preprocessing guidelines followed similar works in dermatology for AI disease detection and telehealth applications [63-65], which aim to objectively show the area of interest for optimized detection and reduce risks of poorly triaged images.

After cropping the images in a 1:1 ratio and before entering the deep learning pipeline, we applied some standard transformations in the data, starting with image resizing. In this paper, we trained, validated, and tested our data set using 5 different models. Notably, 4 of the chosen models (VGG16, Resnet50, EfficientNetV2, and DenseNet169) specified the input images to be resized to 224×224 pixels, and the InceptionV3 model required input images to be resized to 299×299 pixels. Therefore, we proceeded with the image resizing according to each model’s requirements. The last transformation step incorporates normalization of the images, a procedure where the pixel intensity values are standardized across the data set. To help the models generalize better for our data set, we calculated the mean and SD of all images in the data set to use in the normalization process instead of using the ImageNet data set pretrained parameters, inspired by the previous work involving skin disease classification [66].

### Data Set Augmentation

**Figure 2:**
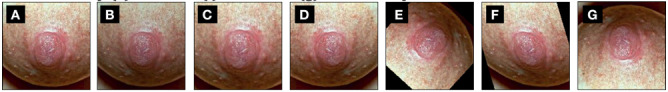
Samples of augmented data: (A) original, (B) brightness, (C) center zoom, (D) horizontal flip, (E) rotation, (F) shear, and (G) vertical flip.

In the process of curating the data set, we recognized that the number of images per class was constrained, given the complexity of gathering images and variability in the clinical features of each class. We implemented data augmentation techniques to mitigate these limitations, reduce the risk of overfitting, and enrich the data set. These techniques artificially expanded the data set by generating realistic transformations of the existing images. We implemented the following 6 data augmentations that were previously used in data sets involving skin lesions [63,67]: center zoom, random rotation, brightness, shear, vertical flip, and horizontal flip. Samples of augmentation are shown in Figure 2. Before data augmentation, our data set consisted of 1078 images. After the augmentation, the data set consisted of 6478 images. The detailed number of samples before and after augmentation is shown in Table 2.

**Figure 3:**
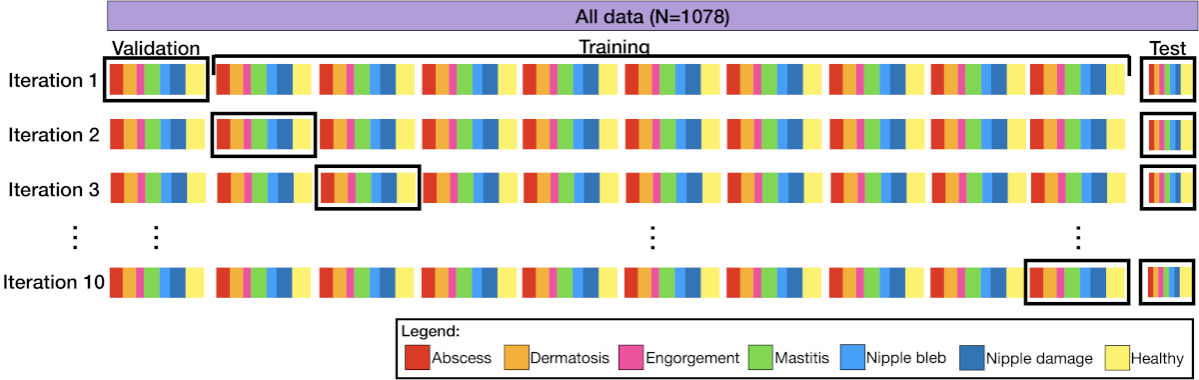
Graphical diagram of stratified k-fold cross-validation on a 7-class data set.

We evaluated our data set before and after data augmentation. In the original data set, the 1000 images were allocated for training and validation, split using stratified k-fold cross-validation [68] with 10 folds. In this process, 90% (900/1000) of the data are used for training and 10% (100/1000) for validation within each fold, as described in Figure 3. The stratified k-fold maintains the proportion of images in each class in both train and validation splits, making sure each fold will be representative of the overall data set. The remaining 78 images were completely excluded from these folds and reserved exclusively for final testing to assess the model’s performance on unseen data. After augmenting the original data set, we expanded it to 6000 images for training and validation. Similarly, we increased our test set to 468 images to maintain consistency with the expanded training data, ensuring the model’s evaluation on unseen examples remains robust.

**Table 2:** Detailed number of samples in the data set.

Data set and classes			Train samples, n		Test samples, n		Train samples (augmented), n		Test samples (augmented), n
**7-class data set**									
	Abscess	108		7		648		42	
	Dermatoses	115		8		690		48	
	Engorgement	55		8		330		48	
	Mastitis	171		9		1026		54	
	Nipple bleb	75		7		450		42	
	Nipple damage	188		9		1128		54	
	Healthy	288		30		1728		180	
**Binary data set**									
	Unhealthy^a^	657		40		3942		240	
	Healthy^a^	343		38		2058		228	

^a^Unhealthy class combines the classes abscess, dermatoses, mastitis, nipple bleb, and nipple damage, while the healthy class combines healthy and engorgement, all from the 7-class data set.

### Feature Extraction

We performed feature extraction using 5 models pretrained on the ImageNet data set. This process helped to reduce the number of computational resources necessary for processing the data set by transforming images into numerical features, without losing relevant information. The models were set to evaluation mode, in which the feature maps are extracted from the final convolutional layers. These maps were then processed through adaptive pooling and flattened into 1D arrays. The extracted features were saved and used as input for the model classifiers.

### Training and Evaluation

As previously mentioned in the AI Algorithms section, a total of 5 CNNs were trained on the data set. We proposed 4 tasks in this study, which evaluates the CNNs in the following data sets: (1) multiclass not augmented, (2) multiclass augmented, (3) binary not augmented, and (4) binary augmented. As described in Table 2, we performed an additional 2 evaluations considering a binary model to assess the models’ capacity to differentiate between healthy and unhealthy images. The unhealthy class consolidates 5 of the previous conditions: abscess, dermatoses, mastitis, nipple bleb, and nipple damage. The healthy class consolidates the original healthy and engorgement conditions. For this binary evaluation, we included engorgement images in the healthy condition because it is not inherently indicative of disease and often resolves without medical intervention. Furthermore, engorgement shares visual characteristics with healthy breast conditions, which might not be distinguishable at an early, nonproblematic stage. All models underwent k-fold cross-validation, where we collected performance metrics from each fold and computed their average. We assessed the models’ performance for the multiclass and binary data sets using the same metrics: accuracy, precision, recall, F_1_-score, and the receiver operating characteristic AUC (ROC-AUC).

## Results

### Overview

We collected 1078 unique breast images from the web and physical resources, 1000 images as part of the training and validation set, and 78 images as part of the testing set. The augmented data set has 6000 images for training and validation and 468 images for testing. In the *Multiclass Image Detection Evaluation* section, we show evaluation results from the multiclass and binary data sets, which we evaluated before and after data augmentation. There was no hyperparameter tuning between each fold, and all models had the same optimizer, learning rate, weight decay, and batch size.

### Multiclass Image Detection Evaluation

**Figure 4:**
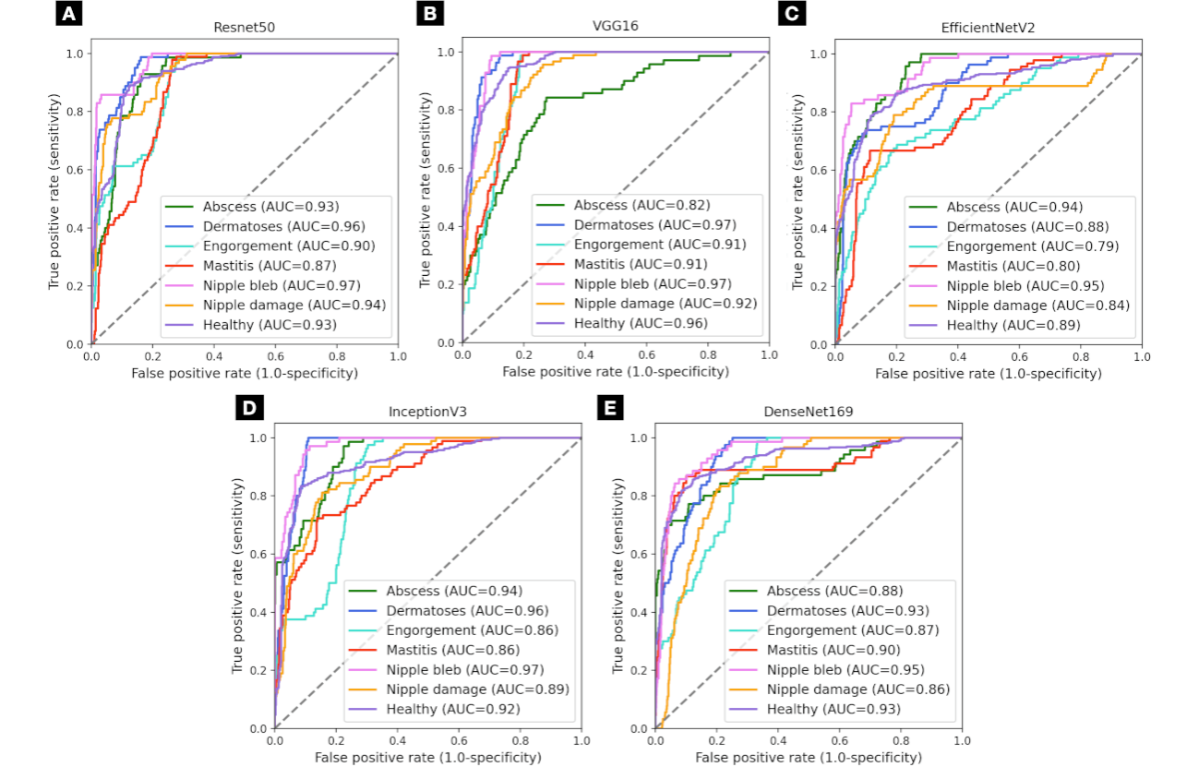
Performance of the 5 convolutional neural networks on the 7-class data set: (A) Resnet50, (B) Visual Geometry Group model with 16 layers (VGG16), (C) EfficientNetV2, (D) InceptionV3, and (E) DenseNet169. AUC: area under the curve.

We evaluated 5 CNNs on their ability to distinguish between healthy and 6 breastfeeding-related issues. Table 3 presents the aggregated evaluation metrics for each model sorted based on the test accuracy. The precision, recall, F_1_-score, and overall area under the ROC-AUC are reported as weighted averages to account for the class imbalance within the data sets, ensuring that each class contributes to the final metric in proportion to its prevalence. For each fold in the cross-validation, a separate test set was used to evaluate the model, and the metrics presented are the mean of these evaluations. The best-performing model was Resnet 50, as it managed to contain the best testing accuracy, followed by VGG16 and EfficientNetV2 on a small performance difference. With a similar weighted average setting, in a one-versus-rest fashion, the models achieved an overall ROC-AUC of 0.934 for VGG16, 0.929 for Resnet50, 0.912 for InceptionV3, 0.908 for Densenet169, and 0.872 for EfficientNetV2. The detailed ROC-AUC per class for each model is shown in Figure 4.

**Figure 5:**
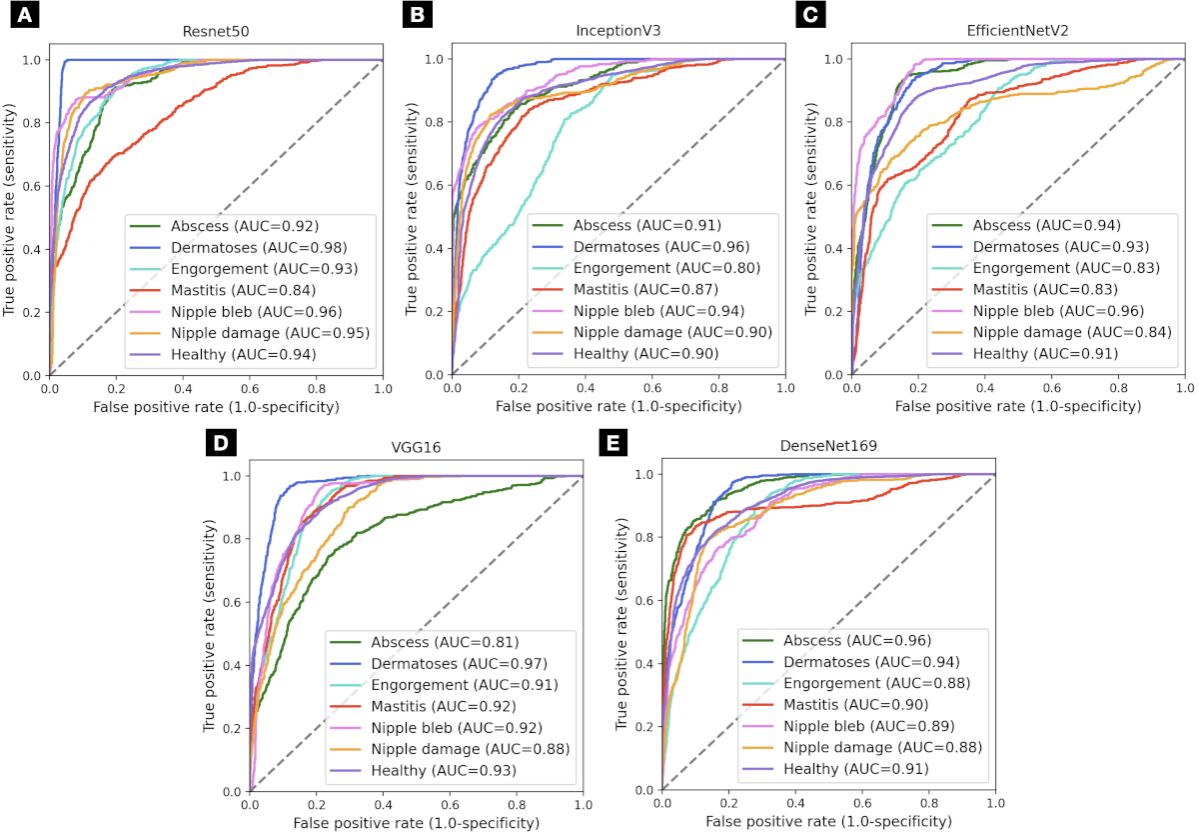
Performance of the 5 convolutional neural networks on the 7-class augmented data set: (A) Resnet50, (B) InceptionV3, (C) EfficientNetV2, (D) Visual Geometry Group model with 16 layers, (E) DenseNet169. AUC: area under the curve.

When applying data augmentation to the multiclass model, we provided a wider variety of images to help the model better generalize from the training data while not altering the original class distribution. In Figure 5 and Table 4, we show the results across the CNNs after data augmentation, where most of the models showed improved metrics, with Resnet50 being the leading model. The models achieved a ROC-AUC of 0.934 for Resnet50, 0.912 for VGG16, 0.909 for Densenet169, 0.898 for InceptionV3, and 0.893 for EfficientNetV2.

**Figure 6:**
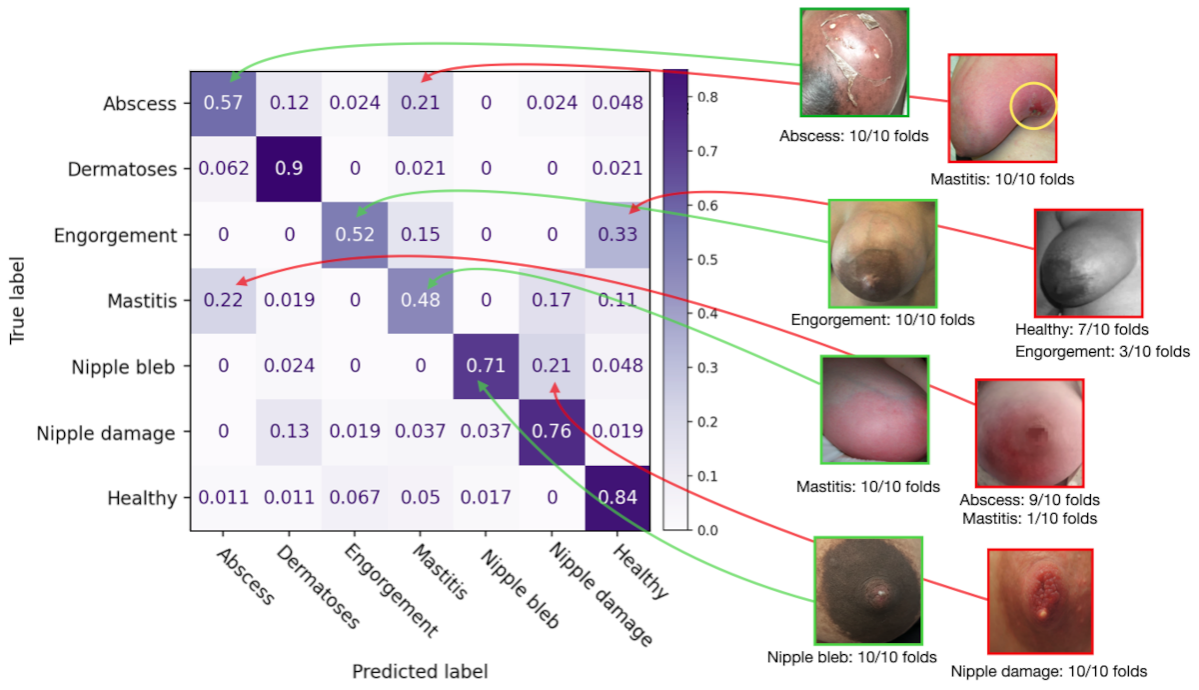
Aggregated confusion matrix for the Resnet50 model for the augmented data set with example images from the augmented data set that were correctly and incorrectly classified across all folders.

Looking into the performance of the best model, the Resnet50 with the augmented data set, we can look closer at the metrics per class of this CNN. Table 5 shows the results for 10-fold cross-validation, in which the model had an overall consistent performance across the iterations. Figure 6 presents the aggregated confusion matrix for the Resnet50 model, in which we consolidated the predictions across all 10 iterations applied to the augmented data set. We achieved this aggregation by taking the median predicted class for each instance over the multiple folds, synthesizing a singular prediction representing the consensus of the model’s behavior across the test set.

Out of the 468 images used in the testing set, the model could correctly classify 341 images. The total images correctly classified by category are as follows: abscess (24/42; accuracy=57%), dermatoses (43/48; accuracy=90%), engorgement (25/48; accuracy=52%), mastitis (26/54; accuracy=48%), nipple bleb (30/42; accuracy=71%), nipple damage (41/54; accuracy=76%), and healthy (152/180; accuracy=84%). The remaining images that were incorrectly classified happened throughout visually similar conditions and the conditions that can precede each other. Table 6 summarizes the selected model’s performance per class on the augmented test set. The model had difficulty categorizing between abscesses, which had false positives on dermatoses and mastitis for 12% (5/42) and 19% (8/42) of the images, respectively. Breast engorgement had false positives on mastitis and healthy breasts for 15% (7/48) and 33% (16/48) of the images, respectively. Mastitis had false positives in abscess (12/54, 22%), nipple damage (9/54, 17%), and healthy breasts (6/54, 11%). About 21% (9/42) of the nipple bleb images were confused as nipple damage.

**Table 3:** Average evaluation metrics for the trained models on the not augmented data set (sorted based on performance).

Data set and model		Training accuracy	Validation accuracy	Test set metrics			
				Accuracy	Precision	Recall	*F*_1_-score
**7-class data set**							
	Resnet50	0.907	0.737	*0.608* ^a^	0.675	*0.623* ^a^	*0.637* ^a^
	VGG16^b^	0.818	0.678	0.604	0.674	0.589	0.600
	EfficientNetV2	0.779	0.626	0.604	0.658	0.582	0.593
	InceptionV3	0.903	0.727	0.574	*0.680* ^a^	0.607	0.622
	DenseNet169	*0.932* ^a^	*0.771* ^a^	0.507	0.659	0.596	0.572

^a^Italicized items represent the best metric.

^b^VGG16: Visual Geometry Group model with 16 layers.

**Table 4:** Average evaluation metrics for the trained models on the augmented data set (sorted based on performance).

Data set and model			Training accuracy		Validation accuracy		Test set metrics						
							Accuracy		Precision		Recall		*F*_1_-score
**7-class augmented data set**													
	Resnet50	0.953		*0.907* ^a^		*0.672* ^a^		*0.717* ^a^		*0.715* ^a^		*0.713* ^a^	
	InceptionV3	0.920		0.844		0.617		0.692		0.637		0.649	
	EfficientNetV2	0.803		0.808		0.602		0.650		0.586		0.5999	
	VGG16^b^	0.755		0.801		0.585		0.644		0.561		0.563	
	DenseNet169	*0.954* ^a^		0.889		0.506		0.639		0.611		0.553	

^a^Italicized items represent the best metric.

^b^VGG16: Visual Geometry Group model with 16 layers.

**Table 5:** Results of 10-fold cross-validation for the augmented data set on Resnet50.

10-fold iterations	Accuracy	Precision	Recall	*F*_1_-score
Iteration 1	0.699	0.705	0.699	0.699
Iteration 2	0.714	0.715	0.714	0.712
Iteration 3	0.709	0.713	0.709	0.709
Iteration 4	0.729	0.730	0.729	0.727
Iteration 5	0.718	0.719	0.718	0.716
Iteration 6	0.733	0.734	0.733	0.730
Iteration 7	0.720	0.722	0.720	0.718
Iteration 8	0.707	0.711	0.707	0.706
Iteration 9	0.707	0.707	0.707	0.705
Iteration 10	0.720	0.715	0.720	0.713

**Table 6:** Summary of the detection results per class: accuracy, precision, recall, F1-score, and support (ie, number of samples per class) using the Resnet50 architecture.

Class	Accuracy	Precision	Recall	*F*_1_-score	Support
Abscess	0.571	0.585	0.571	0.578	42
Dermatoses	0.895	0.729	0.895	0.804	48
Engorgement	0.520	0.641	0.520	0.575	48
Mastitis	0.481	0.481	0.481	0.481	54
Nipple bleb	0.714	0.857	0.714	0.779	54
Nipple damage	0.759	0.683	0.759	0.719	54
Healthy	0.844	0.844	0.844	0.844	180

### Binary Image Detection Evaluation

To improve the accuracy of our clinical predictions and reduce the chances of incorrect results, we simplified our data set of 7 categories to just 2: healthy and unhealthy. The unhealthy category now includes 5 conditions: abscess, dermatoses, mastitis, nipple bleb, and nipple damage. The healthy category now includes the original healthy conditions and engorgement. Engorgement shares many visual similarities with healthy breast conditions, which made it difficult for the multiclass models to identify engorgement accurately. As presented previously, 33% (16/48) of the images of engorgement were classified as healthy. Table 7 presents the aggregated evaluation metrics for 5 models sorted based on the test accuracy.

**Figure 7:**
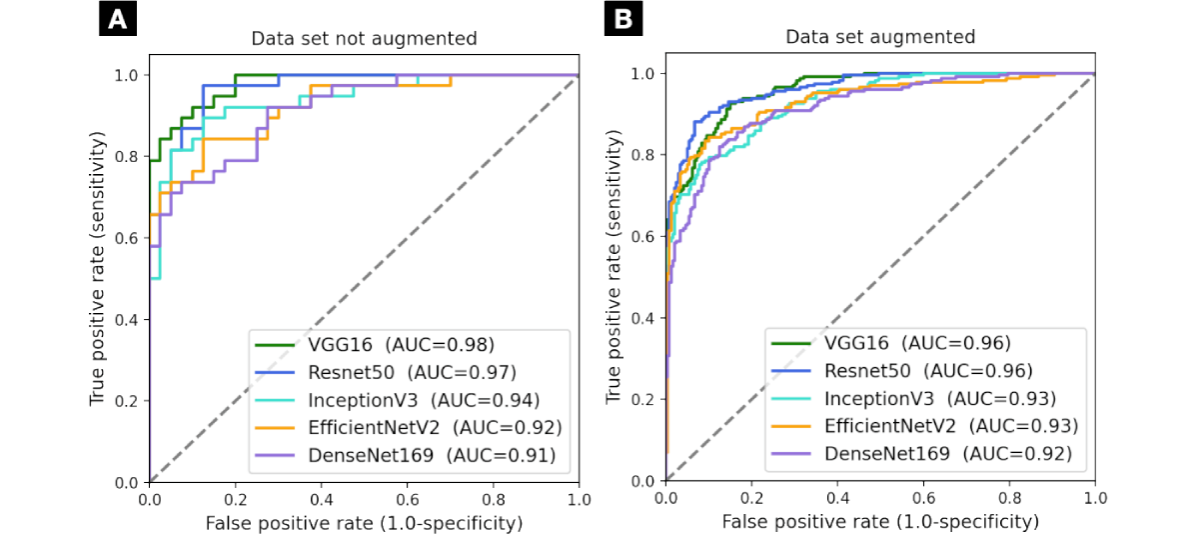
Model performance on the binary data set: (A) without augmentation and (B) with augmentation. AUC: area under the curve; VGG16: Visual Geometry Group model with 16 layers.

The accuracy is reported as a balanced score to address class imbalance, ensuring that each class contributes equally to the final metric. Precision, recall, and F_1_-score are reported for the positive class, with the positive class label specified. For each fold in the cross-validation, we used a separate test set to evaluate the model, and the reported metrics are the average of these evaluations. The best-performing model was the VGG16, which contained the best testing accuracy, followed by Resnet50 and InceptionV3. The models achieved an overall ROC-AUC of 0.977 for VGG16, 0.966 for Resnet50, 0.935 for InceptionV3, 0.921 for EfficientNetV2, and 0.910 for Densenet169. The detailed ROC-AUC for the not augmented and augmented data set is shown in Figures 7A and 7B, respectively.

When applying data augmentation to the binary model, we provided a wider variety of images to help the model better generalize from the training data while not altering the original class distribution. In Table 8, we show the results across the CNNs after data augmentation, where most of the models showed improved metrics, with Resnet50 being the leading model. The models achieved a ROC-AUC of 0.962 for Resnet50, 0.956 for VGG16, 0.931 for EfficientNetV2, 0.929 for InceptionV3, and 0.915 for Densenet169.

**Figure 8:**
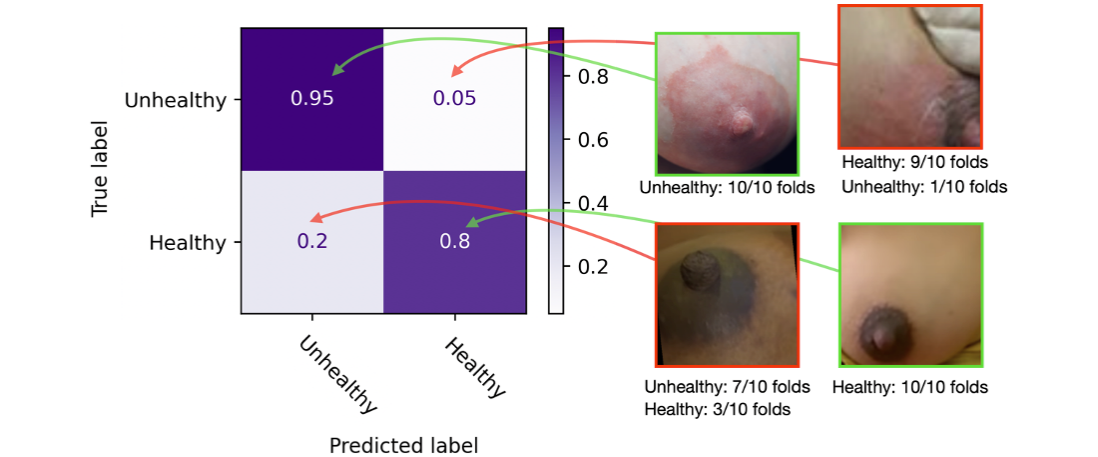
Aggregated confusion matrix for the Resnet50 model for the augmented data set with example images from the augmented data set that were correctly and incorrectly classified across all folders.

Looking into the performance of the best model, the Resnet50 with the augmented data set, we can look closer at the metrics per class of this CNN. Table 9 shows the results for 10-fold cross-validation, in which the model had an overall consistent performance across the iterations. Figure 8 presents the aggregated confusion matrix for the Resnet50 model, in which we consolidated the predictions across all 10 folds applied to the augmented data set. This aggregation was achieved by taking the median predicted class for each instance over the multiple folds, synthesizing a singular prediction representing the consensus of the model’s behavior across the test set.

Out of the 468 images used in the testing set, the model could correctly classify 411 images. The total images correctly classified by category are as follows: unhealthy (228/240; accuracy=95%, precision=83.5%, recall=95% and *F*_1_-score=89%) and healthy (183/228; accuracy=80.3%, precision=94%, recall=80% and *F*_1_-score=86.5%). The remaining images that were incorrectly classified presented redness (ie, for engorgement cases misclassified as unhealthy; 26/228), and incomplete images (ie, too close or nipple and breast not fully visible; 12/228).

**Table 7:** Average evaluation metrics for the trained models on the not augmented binary data set (sorted based on test accuracy).

Data set and model			Training accuracy		Validation accuracy		Test set metrics						
							Accuracy		Precision		Recall	*F*_1_-score	
**Binary data set**													
	VGG16^a^	0.901		0.877		*0.877* ^b^		0.990		*0.760* ^b^			*0.859* ^b^
	Resnet50	0.923		0.872		0.832		0.954		0.715			0.817
	InceptionV3	0.906		0.845		0.838		0.963		0.702			0.812
	EfficientNetV2	0.866		0.831		0.811		*0.991* ^b^		0.629			0.769
	DenseNet169	*0.935* ^b^		*0.880* ^b^		0.761		0.990		0.529			0.688

^a^VGG16: Visual Geometry Group model with 16 layers.

^b^Italicized items represent the best metric.

**Table 8:** Average evaluation metrics for the trained models on the augmented binary data set (sorted based on performance).

Data set and model			Training accuracy		Validation accuracy		Test set metrics						
							Accuracy		Precision		Recall	*F*_1_-score	
**Binary augmented data set**													
	Resnet50	*0.952* ^a^		*0.933* ^a^		*0.877* ^a^		*0.941* ^a^		*0.801* ^a^			*0.865* ^a^
	VGG16^b^	0.877		0.897		0.832		0.941		0.688			0.802
	InceptionV3	0.920		0.893		0.831		0.927		0.715			0.807
	EfficientNetV2	0.885		0.891		0.825		*0.975* ^a^		0.666			0.791
	DenseNet169	0.946		0.927		0.771		0.952		0.570			0.713

^a^Italicized items represent the best metric.

^b^VGG16: Visual Geometry Group model with 16 layers.

**Table 9:** Results of 10-fold cross-validation for the augmented binary data set on Resnet50.

Iteration of 10-fold	Accuracy	Precision	Recall	*F*_1_-score
Iteration 1	0.769	0.948	0.557	0.702
Iteration 2	0.761	0.960	0.531	0.684
Iteration 3	0.791	0.951	0.601	0.737
Iteration 4	0.782	0.970	0.570	0.718
Iteration 5	0.782	0.932	0.596	0.727
Iteration 6	0.778	0.943	0.579	0.717
Iteration 7	0.793	0.928	0.623	0.745
Iteration 8	0.767	0.961	0.544	0.695
Iteration 9	0.778	0.963	0.566	0.713
Iteration 10	0.771	0.969	0.548	0.700

## Discussion

### Principal Findings

The issues that caused model misclassification included (1) wrong positioning of the breast in the image, (2) common visual features in the images between the classes, (3) a lack of variety of images belonging to specific cases in the data set due to variety limitations, and (4) presence of an extraneous object in the frame. Figure 1 presents the correct prediction from the 7 classes.

### Image Quality

**Figure 9:**
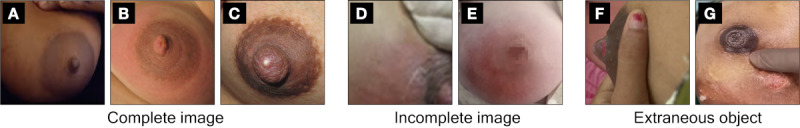
Example images from the testing set. (A), (B), and (C) High-quality images, with a full view of the breast and nipple. (D) Image in which the full breast does not appear, making it hard to classify which condition it belongs to. (E) Although the condition is clear and the full breast is visible, the nipple is pixelated in the photo, altering the original features that the model is not used to. (F) and (G) Partially occluded breasts, and the presence of nail polish in the color of the wound also impacts the model’s performance in those cases. The examples of low-quality data provide details about how to improve data acquisition for future development.

When examining misclassification results in our image data set study, we found many image quality issues that likely contributed to the model’s diminished performance. In the example images from the testing set, Figures 9A-9C demonstrate good image samples that allow a complete evaluation of the breast’s condition and, therefore, can be used for the model’s evaluation. These images fully or almost entirely show the nipple at a distance that allows diagnosis and does not show information about the person’s surroundings or extraneous objects that the model might misinterpret. In Figures 9D and 9E, the main issue in both examples is the lack of nipple or breast presence or only partial presence, making it difficult for the model to assimilate them with breast figures; even if there are signs of mastitis or engorgement in both images, the image is incomplete. For Figures 9F and 9G, the presence of hands or fingers, nail polish, and partially occluded areas with extraneous objects also affects the model interpretation, especially because we did not train the model with such extra components.

Other issues noted in the preprocessing phase were causing issues in training and validation loss as well as false positive and negative detections. For example, having the image of both breasts instead of one affect prediction accuracy, especially in cases where one breast has a different condition compared to the other. The model did not have a large variety of images showing both breasts. Therefore, we improved the training and test results metrics once we separated the breasts into different figures. In addition, we encountered classification problems with extracted images that show some background components, such as clothes surrounding the breast, breast pumps, or segments of the baby’s face or hands. The issues were corrected for these cases by cropping the image to the area of interest. If an object was too similar, such as a hand or a baby, we manually applied blurriness filters in the area and removed saturation so that only the breast is recognizable. Images with low resolution also affect the model’s performance, especially if they are originally smaller than the size determined by the data augmentation algorithm and were stretched later. Some images that belonged to this case and were misclassified had their size manually corrected afterward, and the model properly classified them afterward.

### Visual Similarities Between Conditions

Conditions that present common features and can cause confusion in the diagnosis are mastitis, engorgement, and healthy. Mastitis shows redness throughout the entire breast, showing little skin tone differences and making breasts appear fuller. Some of these features are commonly found in breast engorgement. However, there are fewer signs of intensified redness, sometimes no redness at all, but there may be visible veins and stretched nipples, making them visually similar to healthy ones. Due to the limited availability of images of breast engorgement for a separate class and the fact that engorgement is not necessarily an issue but can become mastitis when not alleviated, the model classified some engorged breasts as mastitis. When we included engorgement in the healthy class for the binary classification, we still got images misclassified as unhealthy, showing how transition conditions should be followed more closely.

This highlights the need for (1) increasing the engorgement data set; (2) working closely with LCs to investigate the need to categorize conditions that can be a problem but indicate false positive cases of more serious issues; and (3) exploring the possibility of using these conditions that have higher errors as a base for following patient condition progression, where there is a transition between conditions for improving or worsening a patient’s situation.

### Lack of Variety of Images Belonging to Specific Cases in the Data Set

**Figure 10:**
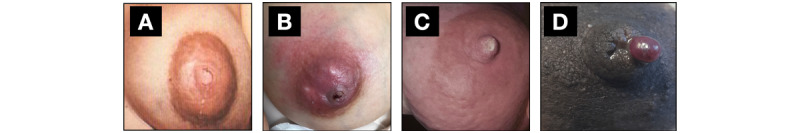
Images incorrectly classified due to data set variety limitations: (A) an engorged breast with an inverted nipple classified as nipple bleb, (B) breast with an abscess but also has nipple damage, (C) breast with granulomatous mastitis classified as nipple damage, and (D) nipple damage classified as nipple bleb.

For the case of Figure 10A, the engorged breast occurs in an inverted nipple, showing its center lighter and misclassifying it as a nipple bleb. Another example of misclassification includes conditions that occur together, which is the case in Figure 10B, showcasing a breast abscess concentrated behind the nipple and with signs of nipple damage. Such an example was one of the very few occurrences of simultaneous conditions in the data set and emphasized the reality that LCs have patients with similar cases, bringing the need to think about systems that (1) recognize multiple conditions or (2) decide between the most severe one for patient priority. Figure 10C is a case of granulomatous mastitis that was classified as nipple damage due to the presence of nipple scarring, highlighting the fewer occurrences of such a specific case in the data set.

In addition, Figures 10C and 10D show breasts in the conditions of engorgement and nipple damage, respectively. For Figure 10D, due to the proximity and nature of the nipple damage with a blood blister, the reflection on the dot suggests that it could be a nipple bleb, also misclassifying the image. These misclassified images with distinct features can also be complex to classify for humans, mainly because some of these conditions rarely occur. Given the nature of the images and the lack of images publicly available with the variety of cases across different skin tones, breasts, and nipple sizes, we believe that working with more images involving rare disorders and providing more data augmentation alternatives can improve the model’s classification significantly. In addition, Figure 10D highlights the issue with image angle and proximity. The picture was taken too close to the breast, having a higher chance of misclassification.

### Limitations

Our findings emphasize the need for improvement in several areas. As demonstrated in our evaluation, naturalistic images captured by users have several image quality issues that can impede the classification system from proper functioning. Thus, future systems must implement a user interface to properly guide parents in taking pictures to input the AI triaging system. This system should provide basic guidelines around how to frame the breast such that no occlusion is present; not use the finger to point out parts of interest; and ensure the camera framing can see the entire breast so that the nipple, areola, and breast tissue are all visible. Previous works explore the importance of implementing guidelines for image assessment of external diseases, such as in dermatology disease assessments, and its benefits for better professional evaluation and higher accuracy in diagnosing conditions [64,65,69]. Guidelines may be implemented as a set of easy instructions, and more advanced systems could provide immediate image quality feedback.

Moreover, our system only uses RGB images to triage breastfeeding-related conditions, not incorporating patient input regarding pain onset, location, symptoms, and pain levels. These are critical data for diagnosing with higher accuracy and providing more effective feedback to patients experiencing breastfeeding-related pain [70]. Furthermore, automating patient responses [71-73] and using large language models [74] can help categorize issues based on their problem description and image inputs, streamlining the care process and ensuring prompt patient attention.

Finally, the most significant limitation of this work is how this evaluation was limited in having a properly balanced data set to help achieve close-to-perfect performance scores from the model. Despite these limitations, we addressed imbalance issues and proved it possible to obtain satisfactory results in detecting and differentiating the conditions we tested.

### Applications and Future Work

This study showcases the potential for high-accuracy breastfeeding-related condition detection to manage postpartum challenges better. In addition, we demonstrate the feasibility of implementing patient support and condition triaging for smartphone-based apps by using deep learning RGB image recognition. The model can be integrated into a telehealth pipeline for postpartum lactation care, helping LCs classify and organize patients based on the severity of their condition or the level of certainty regarding their health concerns. In addition, the system can help track patient disease progression and aid newly qualified LCs by providing faster decision-making support.

The evaluation will serve as a baseline for performing a co-design study with mothers and LCs to evaluate the system requirements regarding data gathering and privacy concerns regarding sensitive data sharing. Understanding the benefits of such a system and recognizing its challenges is essential for building effective tools that will meet patients’ and health care providers’ needs. Furthermore, a comprehensive approach is needed to determine the threshold for flagging a patient as unhealthy in the AI-mediated lactation care system, combining quantitative measures (eg, image detection and pain assessment) with clinical expertise. These improvements will allow this work to compose applications for (1) patient self-assessment tools for actionable feedback for breastfeeding pain, (2) reliably identifying cases that require immediate attention and flagging them for LCs, and (3) enabling timely interventions and improved patient outcomes in lactation care. Future work could envision a fully developed hybrid remote consultation system where patients answer questions for the assessment stage, and images are shared between the patient and provider to visualize the severity of the issue before care is provided. Integrating visual information and pain assessment in remote consultations enhances the diagnostic process and enables LCs to deliver tailored care promptly [75] and help overcome burnout from these professionals.

### Conclusions

This study demonstrates the feasibility of AI-mediated detection of breast conditions for lactating women. We took the first step in this domain by using RGB breast images to triage healthy from unhealthy breasts in mastitis spectrum disease conditions such as nipple blebs, engorgement, abscess, and mastitis; nipple damage caused by poor breastfeeding techniques, breast pumps, and other conditions; and dermatoses caused by a variety of conditions. We implemented 5 distinct CNN models to classify images from 2 different data sets, identifying 7 breast conditions and distinguishing between healthy and unhealthy conditions. The evaluation of the models based on our data set demonstrated the feasibility of using CNNs to classify and intervene with patients who seek remote guidance and management of their symptoms. Although this model’s performance was good, it can be improved by increasing the variety of images and conditions in the data set and implementing the best practices for image posing for proper image classification, leaving significant room for improvement. The feasibility of this work is the initial step toward building tele-lactation services with better data for LCs. We hope our work will inspire future exploration to apply technologies to help lactation support research that can reach more people globally and investigate ideas beyond laboratory settings. This will allow a more comprehensive understanding of breast health for postpartum mothers and empower them to take proactive steps in maintaining their well-being.

## Abbreviations

AI: artificial intelligence
AUC: area under the curve
CNN: convolutional neural network
IBCLC: international board-certified lactation consultant
LC: lactation consultant
RGB: red, green, and blue
ROC-AUC: receiver operating characteristic area under the curve
VGG16: Visual Geometry Group model with 16 layers

## Data Availability

The data sets generated and analyzed during this study are not publicly available due to confidentiality reasons but are available from the corresponding author on reasonable request.
